# Ordered subset expectation maximisation vs Bayesian penalised likelihood reconstruction algorithm in 18F-PSMA-1007 PET/CT

**DOI:** 10.1007/s12149-019-01433-x

**Published:** 2020-01-04

**Authors:** Ewa Witkowska-Patena, Anna Budzyńska, Agnieszka Giżewska, Mirosław Dziuk, Agata Walęcka-Mazur

**Affiliations:** 1 Department of Nuclear Medicine, Military Institute of Medicine, 128 Szaserów St, 04-141 Warsaw, Poland; 2 Affidea Mazovian PET/CT Medical Centre, 128 Szaserów St, 04-349 Warsaw, Poland; 3 Synektik Pharma, Kielce, Poland

**Keywords:** 18F-PSMA-1007, PET/CT, Prostate cancer, Q Clear, OSEM, Reconstruction algorithm

## Abstract

**Background:**

The aim of the study was to compare widely used ordered subset expectation maximisation (OSEM) algorithm with a new Bayesian penalised likelihood (BPL) Q.Clear algorithm in 18F-PSMA-1007 PET/CT.

**Methods:**

We retrospectively assessed 25 18F-PSMA-1007 PET/CT scans with both OSEM and Q.Clear reconstructions available. Each scan was independently reported by two physicians both in OSEM and Q.Clear. SUVmax, SUVmean and tumour-to-background ratio (TBR) of each lesion were measured. Reports were also compared for their final conclusions and the number and localisation of lesions.

**Results:**

In both reconstructions the same 87 lesions were reported. Mean SUVmax, SUVmean and TBR were higher for Q.Clear than OSEM (7.01 vs 6.53 [*p* = 0.052], 4.16 vs 3.84 [*p *= 0.036] and 20.2 vs 16.8 [*p *< 0.00001], respectively). Small lesions (< 10 mm) had statistically significant higher SUVmax, SUVmean and TBR in Q.Clear than OSEM (5.37 vs 4.79 [*p *= 0.032], 3.08 vs 2.70 [*p *= 0.04] and 15.5 vs 12.5 [*p *= 0.00214], respectively). For lesions ≥ 10 mm, no significant differences were observed. Findings with higher tracer avidity (SUVmax ≥ 5) tended to have higher SUVmax, SUVmean and TBR values in Q.Clear (11.6 vs 10.3 [*p *= 0.00278], 7.0 vs 6.7 [*p *= 0.077] and 33.9 vs 26.7 [*p *< 0.00001, respectively). Mean background uptake did not differ significantly between Q.Clear and OSEM (0.42 vs 0.39, *p *= 0.07).

**Conclusions:**

In 18F-PSMA-1007 PET/CT, Q.Clear SUVs and TBR tend to be higher (regardless of lesion localisation), especially for small and highly avid lesions. Increase in SUVs is also higher for lesions with high tracer uptake. Still, Q.Clear does not affect 18F-PSMA-1007 PET/CT specificity and sensitivity.

## Introduction

Ordered subset expectation maximisation (OSEM) is the most commonly used reconstruction algorithm for positron emission tomography (PET) images. In OSEM, data are first divided into subsets and then analysed repetitively during iterations. The higher the number of subsets, the better the qualitative accuracy of images [standardised uptake values (SUVs) of tracer-avid lesions increase]. However, the background noise also increases with each iteration which impedes image visual analysis, especially when it comes to small lesions. Hence, to keep image quality and the number of subsets in balance, the iterative process is stopped prior to complete image convergence, after a limited number of iterations. This may lead to suboptimal image quality, regardless of applied post-reconstruction filters [1–3].

Bayesian penalised likelihood (BPL) reconstruction algorithm named Q.Clear is a new iterative image reconstruction algorithm recently introduced by GE Healthcare (Milwaukee, USA). Q.Clear allows for a full convergence of measured and estimated data due to a point-spread function (PSF) modelling and a noise-controlling penalty term. The penalty term is a function of difference between neighbouring voxels and their sum. It makes low-activity regions appear more smooth and high-activity regions as well as the edges of highly-avid lesions less smooth. This results in more uniform cold backgrounds and higher signal-to-noise ratio of hot, tracer-avid lesions. SUV values measured in Q.Clear should therefore be more accurate and image resolution should be better when compared to OSEM algorithm. The penalty function is controlled by the penalisation factor termed beta which is the only user-dependent variable to the algorithm [1, 4–7].

The aim of our study was to compare SUVs and tumour-to-background ratio (TBR) in OSEM and Q.Clear reconstruction algorithms in 18F-prostate-specific membrane antigen (PSMA)-1007 PET/CT scans. We also assessed the clinical impact of Q.Clear algorithm and checked whether it alters the general impression of OSEM-reconstructed report and upgrades or downgrades the disease staging.

## Materials and methods

### Patients

Between February 1st, 2019 and November 30th, 2019, 61 18F-PSMA-1007 PET/CT scans were performed at the Military Institute of Medicine (Warsaw, Poland). Both OSEM and Q.Clear reconstruction were available for 25 scans of 25 patients (in our institution routinely only the OSEM reconstruction is performed). Indications for 18F-PSMA-1007 PET/CT were: biochemical relapse (BCR) of prostate cancer (PCa), treatment response evaluation, PCa suspicion and PCa primary staging. Patients’ characteristics are given in Table 1.

**Table 1:** Patients’ characteristics

Characteristic	Value
Number of patients	25
Age	
Mean ± SD	65.9 ± 8.5 years
Median (range)	66.0 (47.0–85.0) years
Indication for PET/CT	Biochemical relapse (*n* = 15, 60%)
	Suspected PCa (*n* = 6, 24%)
	Treatment response evaluation (*n* = 3, 12%)
	Primary staging (*n* = 1, 4%)
PSA level	
Mean ± SD	2.34 ± 3.9 ng/ml
Median (range)	0.65 (0.02–14.0) ng/ml
Administered activity	
Mean ± SD	312.8 ± 13.7 MBq
Median (range)	310.0 (295.0–344.0) MBq
Uptake time	
Mean ± SD	61.1 ± 1.5 min
Median (range)	60.0 (60.0–64.0) min

*SD* standard deviation, *PCa* prostate cancer, *PSA* prostate-specific antigen, *MBq* megabecquerels

All procedures performed in this retrospective observational study were in accordance with national regulations and with the 1964 Helsinki declaration and its later amendments. Informed consent was obtained from all individual participants included in the study. Military Institute of Medicine in Warsaw, Poland does not require Ethics Committee approval for retrospective observational studies.

### Radiotracer synthesis

18F-PSMA-1007 was made with a Trasis AiO (Ans, Belgium) synthesizer. 18F-fluoride was produced in Siemens Eclipse (Knoxville, USA) cyclotron by bombarding enriched 18O-water with protons. It was then collected at anion exchange cartridge (QMA) and released by tetrabutylammonium hydroxide (TBA-HCO_3_) eluent to reaction vial where residual traces of water were evaporated at 130 °C for 8 min. Then, PSMA-1007 precursor (ABX, Radeberg, Germany) dissolved in 2 ml of dimethyl sulphoxide (DMSO) was added to the dried complex. Fluorination reaction was processed at 105 °C for 5 min. During labelling, two cleaning cartridges (C18ec and PS-H) were conditioned by rinsing with 5% ethanol (EtOH) in water for injection (WFI), EtOH and again 5% EtOH in WFI. Crude product was trapped on cartridges and rinsed with 5% EtOH in WFI to remove side-products. Product was eluted from cartridges by 30% EtOH to the end vial by 0.22 µm sterilizing filter and then diluted by phosphate buffered saline. In the end, quality control was performed.

### Imaging protocol

PET/CT imaging was performed with a hybrid PET/CT system Discovery 710 (GE Healthcare, Milwaukee, USA). Scans were obtained approximately 60 min after injection of 18F-PSMA-1007. First, a scout view and a non-contrast-enhanced low-dose spiral 64-slice CT scan was performed for attenuation correction of PET and for anatomic localisation. CT scan was acquired with a tube voltage of 140 kV in the helical mode with a Smart/Auto mA (range 40–120 mA). The X-ray tube rotation time was 0.6 s. The pitch and table speed were 0.984:1 and 39.37 mm/rot, respectively. The helical thickness was 3.75 mm. For standard type of reconstruction, the slice thickness was 1.25 mm. The GE ASIR (Adaptive Statistical Iterative Reconstruction) with the level of 20% was used to reduce patient radiation dose from CT scans.

Following CT, top-of-the-head to mid-thigh three-dimensional PET was acquired. For each bed position (15.7 cm with 23% bed overlap), a 3-min long acquisition time was used. The emission data were corrected for geometrical response, detector efficiency, system dead time, random coincidences, scatter and attenuation.

Attenuation corrected images were reconstructed with two iterative algorithms—OSEM and Q.Clear. OSEM was set for 3 iterations/18 subsets and a filter cut-off of 5.5 mm. The matrix size was 256 × 256. Time of flight (TOF) (GE VUE Point FX) and a resolution recovery algorithm (GE SharpIR) were on. Q.Clear images were reconstructed with default producer settings: beta value 350, no post filtering and TOF disabled. Q.Clear algorithm uses PSF modelling so GE SharpIR was automatically on (without off option).

### Image analysis

Advantage Workstation (GE Healthcare, Milwaukee, USA) was used for image analysis. Each scan was evaluated both in OSEM and Q.Clear reconstruction independently by two physicians experienced in 18F-PSMA-1007 PET/CT. All tracer-avid lesions with uptake higher than the background and not associated with physiological 18F-PSMA-1007 uptake were considered malignant and reported. For each lesion, mean and maximum SUV (SUVmean and SUVmax) values were measured and tumour-to-background ratios (TBR) were calculated. To measure SUVs, rectangular regions of interest (ROI) were drawn around areas with focally increased uptake in axial slices and then adapted to 3-D volume of interest. To calculate TBR, lesion SUVmax was divided by SUVmean of the background (pelvic fat tissue).

Lesions were also divided and compared according to their size (greatest diameter < 10 mm vs ≥ 10 mm), SUVmax value in OSEM reconstruction (< 5 vs ≥ 5) and localisation (bone, lymph nodes and soft tissue). Diameter was measured on CT scans. Lesions visible on PET scans only (CT-negative) were not taken into account in size (< 10 mm vs ≥ 10 mm) comparison.

### Statistical analysis

Statistica 13 software (StatSoft Polska Sp. z o. o., Cracow, Poland) was used for statistical analysis. Descriptive analysis was performed by calculating mean, median, standard deviation and range. Paired samples were compared using the Wilcoxon signed-rank test and the sign test. A *p* value of < 0.05 was considered significant.

## Results

No adverse effects were observed after injection of 18F-PSMA-1007. Patients did not report any alarming symptoms.

**Fig. 1:**
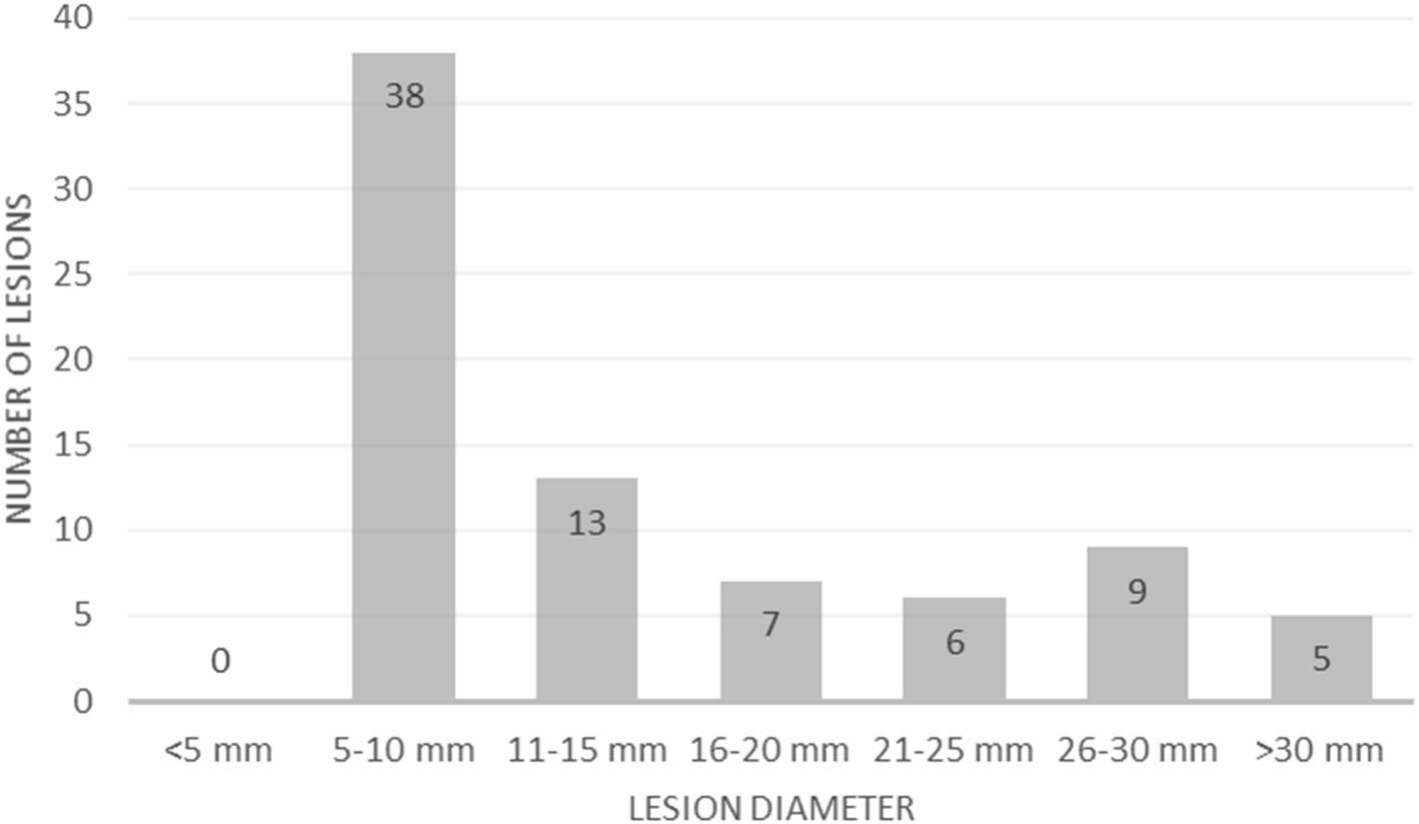
Reported lesions grouped, according to their size

In both reconstructions, the two reporting physicians described the same 87 lesions: 30 (34%) were in bone, 29 (33%) in lymph nodes and 28 (32%) in soft tissue (in the prostate gland, seminal vesicles and prostate bed; there was also one metastasis in the liver). The lesions’ diameter ranged from 6 to 61 mm. The mean diameter was 14.9 mm in the bones, 18.0 mm in the lymph nodes and 14.6 mm in soft tissues (Fig. 1). The mean values of SUVmax, SUVmean and TBR were higher in Q.Clear than OSEM in all three of the above localisations yet only differences in TBR were statistically significant (Table 2). Seventy-eight lesions (90%) were visible in PET and CT, nine lesions (10%) were CT-negative. Report conclusions did not differ between the two algorithms and the two reporting physicians.

**Table 2:** Mean values of SUVmax, SUVmean and TBR, according to the lesion localisation

	OSEM			Q.Clear		
	Bone	Lymph nodes	Soft tissue	Bone	Lymph nodes	Soft tissue
SUVmax	5.40	6.88	7.39	5.43	7.23	8.47
SUVmean	3.18	4.01	4.38	3.12	4.14	5.30
TBR	12.46	17.34	20.95	13.57	20.07	27.43

*OSEM* ordered subset expectation maximisation, *SUVmax* maximum standardised uptake value, *SUVmean* mean standardised uptake value, *TBR* tumour-to-background ratio

**Fig. 2:**
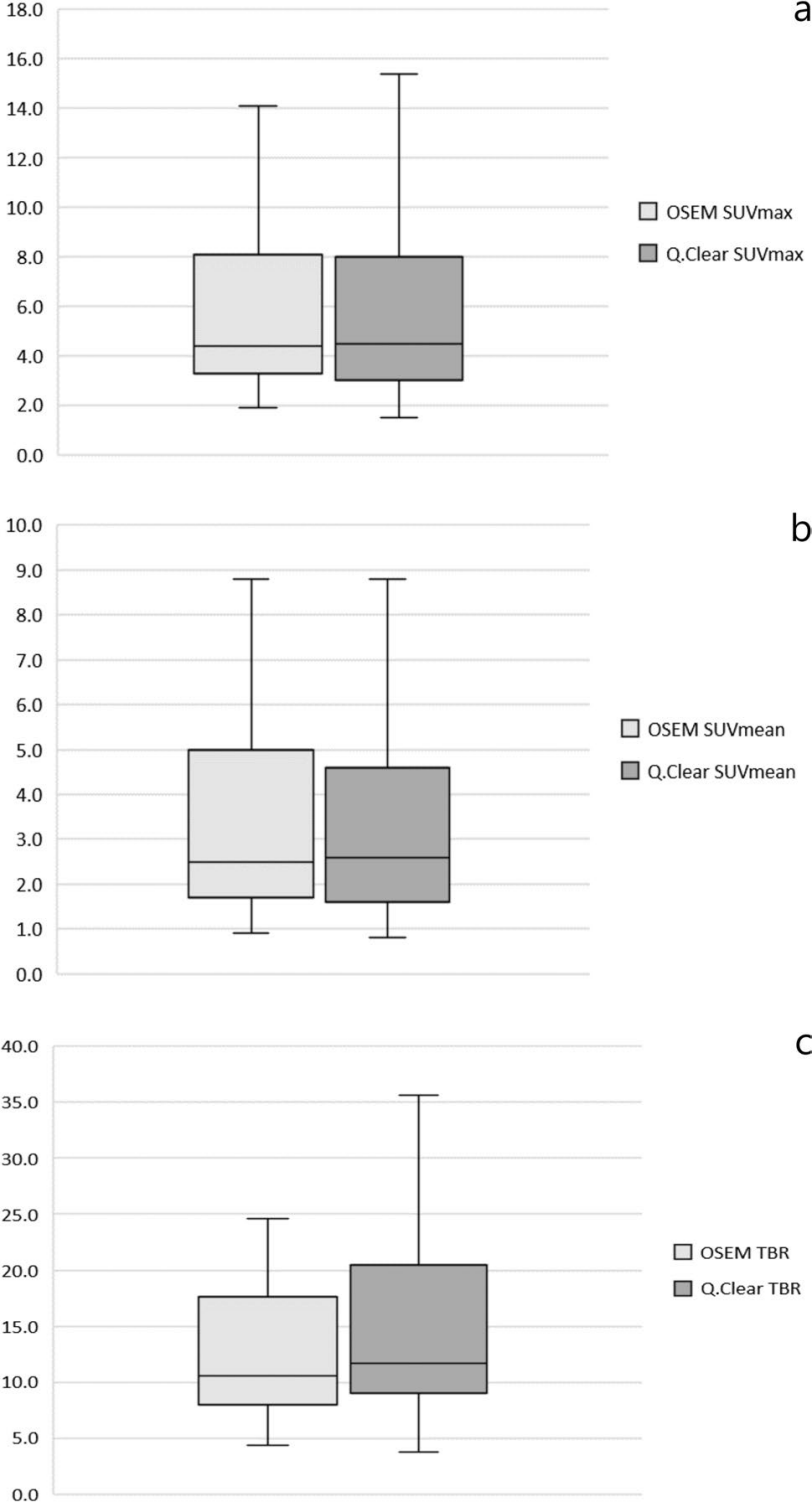
SUVmax (**a**), SUVmean (**b**) and TBR (**c**) variation of all assessed lesions. The first and third quartile are represented by the bottom and top side of the box, respectively. Median is the line inside the box. Whiskers extend to the minimum and the maximum values of the data set (without outliers)

Mean values of SUVmax, SUVmean and TBR were higher in Q.Clear reconstruction algorithm than in OSEM and measured 7.01 vs 6.53 (*p *= 0.052), 4.16 vs 3.84 (*p *= 0.036) and 20.2 vs 16.8 (*p *< 0.00001), respectively (Fig. 2).

**Fig. 3:**
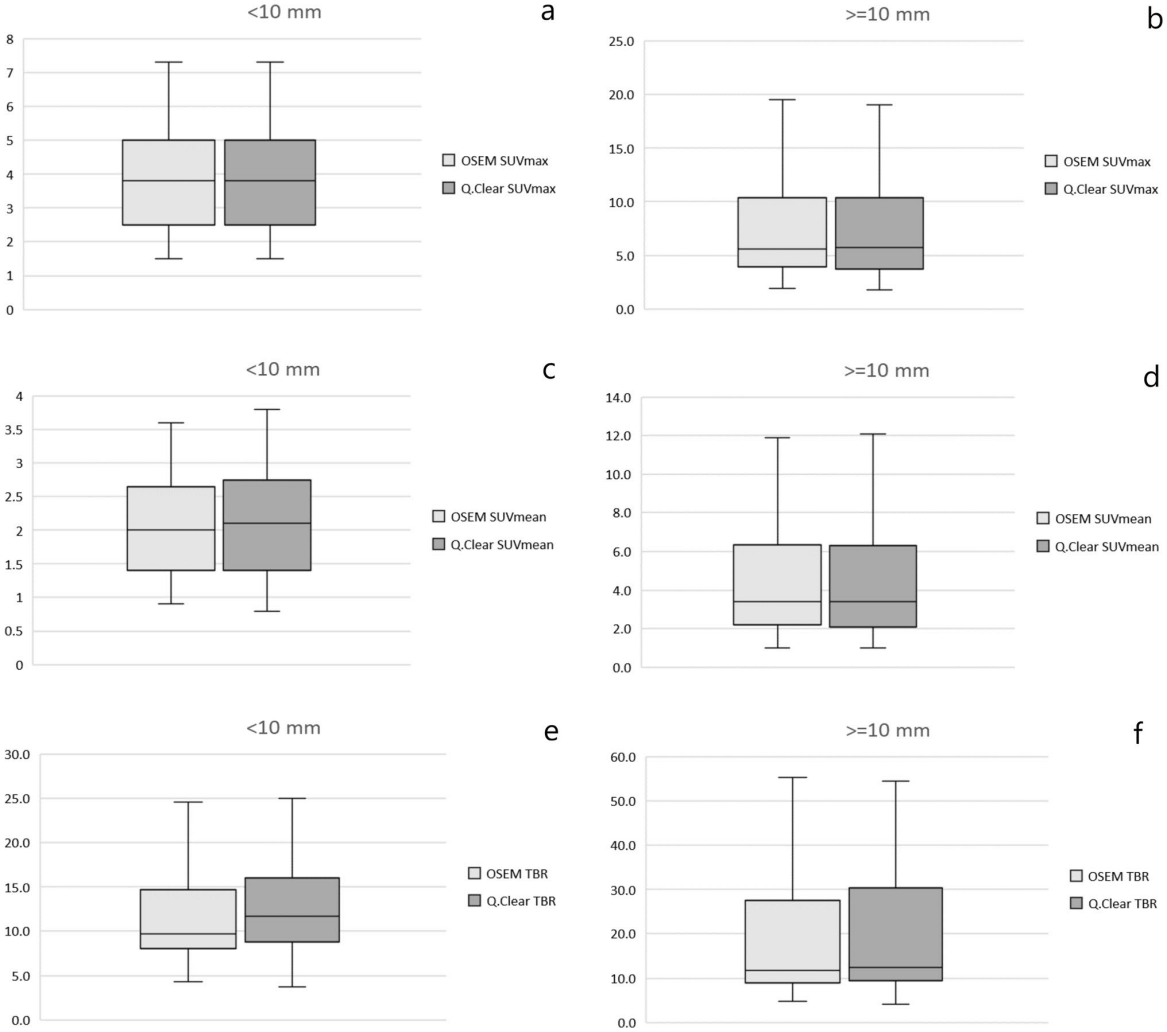
SUVmax, SUVmean and TBR variation based on the size of lesions: < 10 mm (**a**, **c**, **e**) and ≥ 10 mm (**b**, **d**, **f**). The first and third quartile are represented by the bottom and top side of the box, respectively. Median is the line inside the box. Whiskers extend to the minimum and the maximum values of the data set (without outliers)

Mean values of SUVmax, SUVmean and TBR were also higher for Q.Clear than OSEM when the lesions were divided according to their size. For small lesions (< 10 mm, *n* = 29, 30%), they were 5.37 vs 4.79 (*p *= 0.032), 3.08 vs 2.70 (*p *= 0.04) and 15.5 vs 12.5 (*p *= 0.00214), respectively. For lesions ≥ 10 mm (*n* = 49, 70%) they were as follows: 8.36 vs 7.95 (*p *= 0.069), 5.06 vs 4.75 (*p *= 0.081) and 24.3 vs 20.5 (*p *< 0.00001) (Fig. 3).

**Fig. 4:**
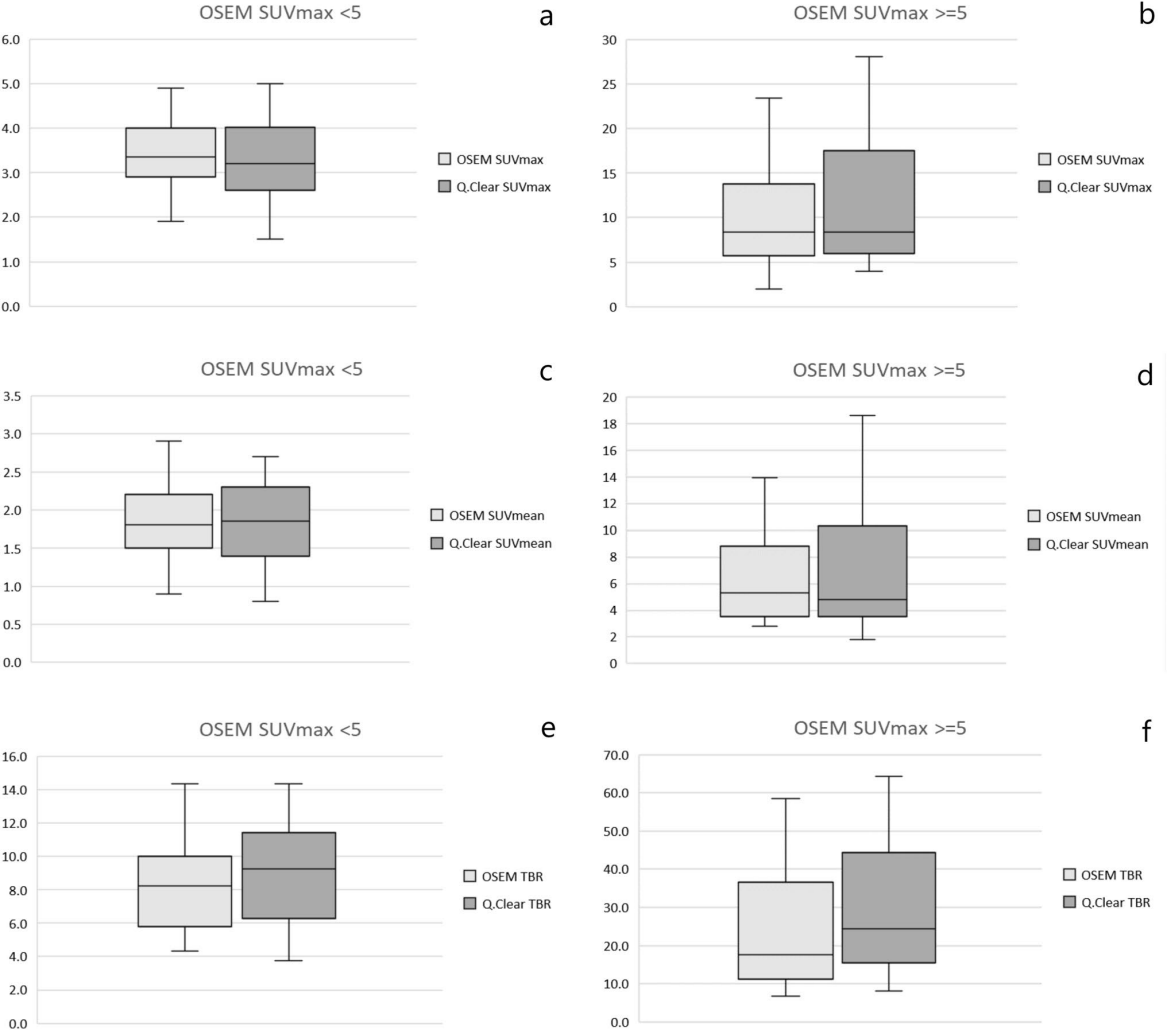
SUVmax, SUVmean and TBR variation based on the avidity of lesions measured in OSEM: < 5 (**a**, **c**, **e**) and ≥ 5 (**b**, **d**, **f**). The first and third quartile are represented by the bottom and top side of the box, respectively. Median is the line inside the box. Whiskers extend to the minimum and the maximum values of the data set (without outliers)

When lesions were divided according to 18F-PSMA-1007 avidity, findings with SUVmax < 5 in OSEM (*n* = 48, 55%) tended to have similar OSEM and Q.Clear mean SUVmax (3.4 vs 3.3, *p *= 0.11) and SUVmean (1.9 vs 1.9, *p* = 0.47). TBR values were insignificantly higher for Q.Clear—9.1 vs 8.6, *p *= 0.10. On the other hand, mean Q.Clear SUVmax, SUVmean and TBR were higher than OSEM when lesions with SUVmax ≥ 5 (*n* = 39, 45%) were considered. They measured 11.6 vs 10.3 (*p *= 0.00278), 7.0 vs 6.7 (*p *= 0.077 and 33.9 vs 26.7 (*p *< 0.00001), respectively (Fig. 4).

Mean background uptake (SUVmean) for OSEM and Q.Clear was 0.42 vs 0.39 (*p *= 0.07), respectively.

## Discussion

Here we presented the results of OSEM vs Q.Clear reconstruction algorithm comparison in 18F-PSMA-1007 PET/CT. To our knowledge, this is the first analysis of the two algorithms in PET with this particular radiotracer.

In our study, we found that mean SUVmax, SUVmean and TBR values in 18F-PSMA-1007 PET/CT were generally higher for Q.Clear reconstruction algorithm than OSEM, regardless of the lesion localisation. The exception was the lesions with OSEM SUVmax values < 5 where OSEM reconstruction yielded similar values than Q.Clear. However, some of the differences were not statistically significant.

**Fig. 5:**
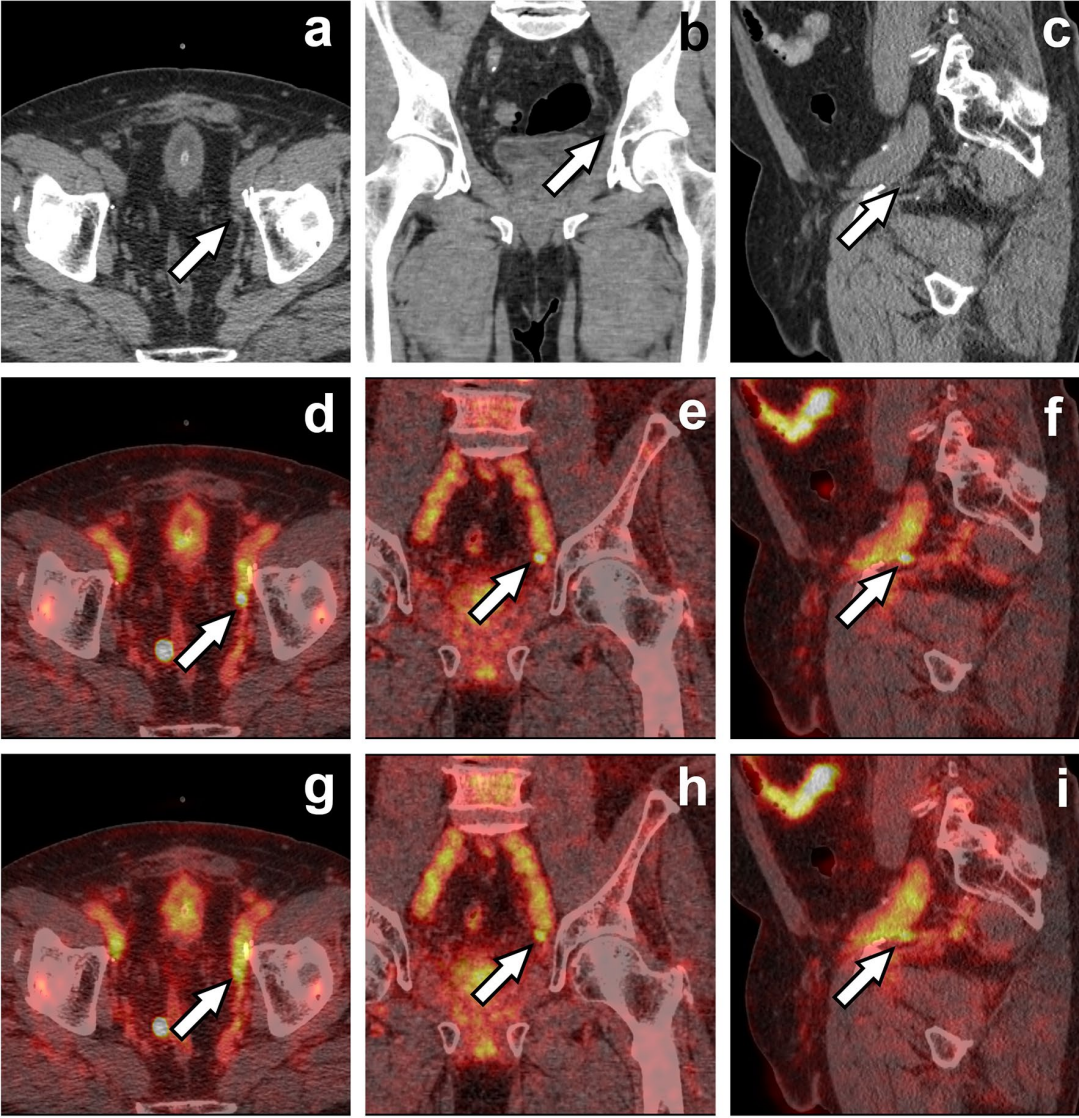
A 60-year-old prostate cancer patient after radical prostatectomy and pelvic radiation beam therapy with biochemical relapse (PSA 0.72 ng/ml). Axial, coronal and sagittal CT (**a**–**c**), Q.Clear fused PET/CT (**d**–**f**) and OSEM fused PET/CT (**g**–**i**) images are presented. White arrow indicates a tracer-avid left internal iliac node (6 mm in short axis) which seems more distinct on Q.Clear images (**d**–**f**). The node also had higher Q.Clear than OSEM values of SUVmax (5.3 vs 4.9), SUVmean (3.3 vs 2.8) and TBR (25.0 vs 17.7)

Matti et al. showed that Q.Clear is more efficient than OSEM + PSF + TOF to determine SUVmax and SUVmean values in 18F-fluorodeoxyglucose (FDG) PET. The authors also reported that Q.Clear superiority is most evident in small lesions [1, 8]. Similarly, we found statistically significant differences of SUVmax and SUVmean values for lesions < 10 mm (Fig. 5). Findings ≥ 10 mm did not differ significantly (except for TBR).

Q.Clear yields greatest increases in SUVmax and SUVmean values for findings with high tracer avidity (SUVmax ≥ 10) [1]. Teoh et al. have also reported that Q.Clear algorithm yields smaller SUVmax increments in lesions with low tracer uptake (below background) [9]. In our study, findings with SUVmax ≥ 5 had higher Q.Clear vs OSEM differences than lesions with SUVmax < 5. Mean SUVmax values of the former ones (< 5) were similar in Q.Clear and OSEM.

It has been shown that the value of beta in Q.Clear algorithm affects the SUVmax and SUVmean values so that the higher the beta value, the lower the SUVs [10]. To our knowledge, no studies determining the optimal beta value for 18F-PSMA-1007 have been published so far. Hence, in our study a default value of 350 suggested by the producer was used. The optimal beta values depend on the type of radiopharmaceutical and the anatomical region scanned. In 18F-FDG PET imaging, the optimal beta for torso scans is between 300 and 400 and between 100 and 200 for brain scans [4]. The optimal beta values for some prostate cancer-targeting tracers have been reported as: 400–550 for 68Ga-PSMA, 400–550 for 18F-fluorocholine and 300 for 18F-flucyclovine [5, 11, 12].

Q.Clear reconstruction algorithm did not increase 18F-PSMA-1007 PET/CT sensitivity or specificity (the number and characterisation of findings were the same for both OSEM and Q.Clear) which is in accordance with the literature. The decrease in background uptake in Q.Clear was also insignificant which is in line with previous reports that BPL increases signal to background ratio by increasing SUVs of tracer-avid lesions rather than by reducing background uptake [1].

The main limitation of the study was a relatively small number of assessed lesions. It may be the cause for the lack of statistically significant differences in some comparisons. It may also be because of disabled TOF function in Q.Clear which underestimated SUVs. The impact of TOF status on Q.Clear images in 18F-PSMA-1007 PET is now under investigation in our institution.

## Conclusions

Q.Clear reconstruction algorithm seems valuable in 18F-PSMA-1007 PET/CT. In Q.Clear, SUVs and TBR tend to be higher than in OSEM (regardless of the lesion localisation), especially for small lesions. Increase in SUVs is also higher for lesions with high tracer uptake. Still, Q.Clear does not affect 18F-PSMA-1007 PET specificity and sensitivity.
